# Relationship between optical coherence tomography sector peripapillary angioflow-density and Octopus visual field cluster mean defect values

**DOI:** 10.1371/journal.pone.0171541

**Published:** 2017-02-02

**Authors:** Gábor Holló

**Affiliations:** Department of Ophthalmology, Semmelweis University, Budapest, Hungary; Roskamp Institute, UNITED STATES

## Abstract

**Purpose:**

To compare the relationship of Octopus perimeter cluster mean-defect (cluster MD) values with the spatially corresponding optical coherence tomography (OCT) sector peripapillary angioflow vessel-density (PAFD) and sector retinal nerve fiber layer thickness (RNFLT) values.

**Methods:**

High quality PAFD and RNFLT images acquired on the same day with the Angiovue/RTVue-XR Avanti OCT (Optovue Inc., Fremont, USA) on 1 eye of 27 stable early-to-moderate glaucoma, 22 medically controlled ocular hypertensive and 13 healthy participants were analyzed. Octopus G2 normal visual field test was made within 3 months from the imaging.

**Results:**

Total peripapillary PAFD and RNFLT showed similar strong positive correlation with global mean sensitivity (r-values: 0.6710 and 0.6088, P<0.0001), and similar (P = 0.9614) strong negative correlation (r-values: -0.4462 and -0.4412, P≤0.004) with global MD. Both inferotemporal and superotemporal sector PAFD were significantly (≤0.039) lower in glaucoma than in the other groups. No significant difference between the corresponding inferotemporal and superotemporal parameters was seen. The coefficient of determination (R^2^) calculated for the relationship between inferotemporal sector PAFD and superotemporal cluster MD (0.5141, P<0.0001) was significantly greater than that between inferotemporal sector RNFLT and superotemporal cluster MD (0.2546, P = 0.0001). The R^2^ values calculated for the relationships between superotemporal sector PAFD and RNFLT, and inferotemporal cluster MD were similar (0.3747 and 0.4037, respectively, P<0.0001).

**Conclusion:**

In the current population the relationship between inferotemporal sector PAFD and superotemporal cluster MD was strong. It was stronger than that between inferotemporal sector RNFLT and superotemporal cluster MD. Further investigations are necessary to clarify if our results are valid for other populations and can be usefully applied for glaucoma research.

## Introduction

For several decades vascular dysregulation and unstable perfusion of the optic nerve head and the peripapillary retina have been considered as risk factors for the development and progression of open-angle glaucoma [1–5]. In contrast to retrobulbar perfusion, global retinal oxygenation and ocular perfusion pressure, which can be measured and calculated, local microcirculation abnormalities in and around the disc are very difficult to measure noninvasively [1–7]. This is one reason why causal relationship between localized vascular dysfunction or decreased vessel density in the optic nerve head and the peripapillary retina and the development of spatially corresponding glaucomatous structural damage is difficult to establish. Optical coherence tomography (OCT) angiography is a non-invasive technology, which was recently developed to measure perfusion in various layers of the retina in the macula, the optic nerve head and the peripapillary area, respectively [8–16]. Using Angiovue OCT angiography it has been shown that disc and peripapillary vessel density (which reflects perfusion and not the anatomical density of the vessels) decreases in glaucoma, and that the reduction is related to global glaucomatous visual field deterioration, retinal nerve fiber layer thickness (RNFLT), macular inner retina thickness and the glaucoma stage [8, 9, 11, 16–19]. These functional results confirm the results of the earlier anatomical investigations which found a strong relationship between retinal nerve fiber quantity and the spatially corresponding vessel quantity in glaucoma [20, 21]. Recently, using the Angiovue/RTVue-XR Avanti OCT (Optovue Inc., Fremont, USA) and the Optovue 2015.100.0.33 software version we have shown that sector peripapillary angioflow vessel-density (PAFD, expressed in % of the measured area) measurements show favorable short-term and long-term reproducibility in glaucoma and ocular hypertension [17]; in the radial peripapillary capillaries (RPC) layer, which reflects the retinal nerve fiber layer, sector PAFD values show strong to very strong positive correlation with the corresponding sector RNFLT values [17]; sector PAFD can identify decreased peripapillary perfusion early, even prior to the development of clinically significant RNFLT thinning and visual field deterioration [14]; and decreased PAFD spatially corresponds with the damaged retinal nerve fiber bundles [14].

**Fig 1:**
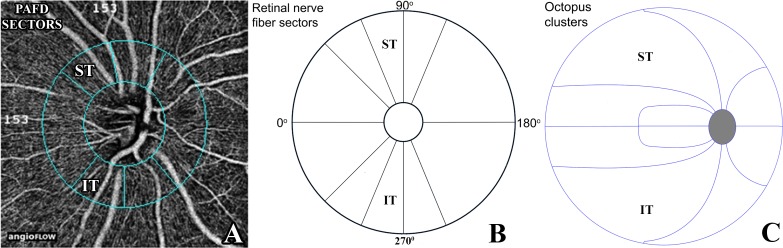
**Peripapillary sectors provided by the Optovue 2015.100.0.33 software version for peripapillary vessel density measurement with the Angiovue OCT (A), the spatially corresponding retinal nerve fiber layer thickness sectors (B), and the spatially corresponding Octopus perimeter clusters (C)** Each of the 10 retinal nerve fiber layer sectors (B) spatially corresponds to a cluster (C).PAFD, peripapillary angioflow-density; ST, superotemporal; IT, inferotemporal;

In glaucoma structural damage is often localized in early to moderate disease stages. Recently we have shown strong positive relationship between narrow sector RNFLT and Octopus visual field cluster sensitivity values [22], and moderate-to-strong negative relationship between 10 sector RNFLT values and the corresponding 10 Octopus visual field cluster mean defect (cluster MD) values [23]. We found a particularly strong relationship between inferotemporal RNFLT and the corresponding superotemporal cluster MD [23]. In the current investigation we evaluated the relationship between sector PAFD and Octopus visual field cluster MD values for those sector—cluster pairs which have been established in our previous investigations and are available with the software version used by us (the inferotemporal sector—superotemporal cluster pair; and the superotemporal sector—inferotemporal cluster pair, Fig 1). In order to investigate whether sector PAFD and sector RNFLT decrease parallel across the glaucoma spectrum we compared the relationship of sector PAFD and sector RNFLT with the corresponding visual field cluster in a cross-sectional study using healthy normal, under treatment ocular hypertensive and glaucoma eyes.

## Materials and methods

### Participants and protocol

The research protocol was approved by the Institutional Review Board for Human Research of Semmelweis University, Budapest. Written informed consent was obtained from all participants before enrolment. All applicable institutional and governmental regulations concerning the ethical use of human volunteers were followed. All participants were white Europeans participating in a long-term imaging study in the Glaucoma Center of the Semmelweis University in Budapest. OCT angiography and retinal nerve fiber layer imaging were conducted prospectively between June and December 2015. During the study visit high quality OCT angiography measurements of the optic nerve head and peripapillary retina, and high quality imaging for RNFLT measurement were made. The participants underwent determination of the current best corrected visual acuity, evaluation of the central 30-degree visual field using Octopus perimetry, and a detailed ophthalmological examination.

The study population comprised 13 healthy eyes of 13 healthy subjects (with mean defect [MD] less than 2 dB using Octopus perimetry normal strategy, Loss Variance [LV] less than 6 dB^2^, and no significantly decreased test point sensitivity value) and untreated intraocular pressure consistently below 22 mmHg; 22 under treatment ocular hypertensive (OHT) eyes of 22 OHT patients with normal optic nerve head and visual field (with MD less than 2 dB using Octopus perimetry normal strategy, Loss Variance [LV] less than 6 dB^2^, and no significantly decreased test point sensitivity value) and untreated intraocular pressure consistently above 21 mmHg in both eyes; and 27 under treatment stable primary open-angle glaucoma eyes of 27 primary-open angle glaucoma patients characterized with untreated intraocular pressure consistently above 21 mmHg, glaucomatous neuroretinal rim loss and reliable and reproducible visual field defect typical for glaucoma (inferior and/or superior paracentral or arcuate scotomas, nasal step, hemifield defect or generalized depression with Octopus perimetry MD higher than 2 dB using normal strategy). One eye per participant (the eye with a higher PAFD image quality score) was analyzed. The demographics are shown in Table 1.

**Table 1:** Demographics of the study eyes.

	Healthy eyes (n = 13)	OHT eyes (n = 22)	Glaucoma eyes (n = 27)
Age (years; median, quartiles)	62.0	61.5	62.0
	54.0 to 73.0	47.0 to 70.0	51.0 to 68.0
Spherical equivalent (Diopters; median, quartiles)	-0.50	-0.75	0.0
	-2.00 to 0.50	-2.50 to 0.00	-2.50 to 1.50
Best corrected visual acuity (median, quartiles)	1.0	1.0	1.0
	1.0 to 1.0	1.0 to 1.0	1.0 to 1.0
Pupil diameter during perimetry (mm; median, quartiles)	4.6	4.7	4.9
	3.4 to 5.6	4.2 to 5.4	4.4 to 5.5
Global mean sensitivity, normal strategy (dB; median, quartiles)	26.4	26.9	20.7
	26.1 to 28.0	26.0 to 28.0	12.2 to 24.4
Global mean defect*, normal strategy (dB; median, quartiles)	-0.3	-0.1	5.0
	-0.8 to 0.3	-0.7 to 0.5	1.0 to 10.7
Total peripapillary RNFLT (μm; mean ±SD)	93.1± 10.0	92.7 ± 8.2	70.7 ± 12.8

*In Octopus perimetry pathological mean deviation (MD) values are positive numbers

OHT, ocular hypertension

### Determination of peripapillary angioflow-density

We used a new and commercially not yet released software (the Optovue 2015.100.0.33 software version, Optovue Inc., Fremont, CA, USA) to measure PAFD (expressed in % of the measured area) via undilated pupil in the total peripapillary measurement area and in each of the 6 peripapillary sectors (Fig 1) in 2 different layers. In the current investigation measurements made for the inferotemporal and superotemporal sectors and the total peripapillary area in the RPC layer were used. The RPC layer reflects the retinal nerve fiber layer with the Angiovue OCT in which a uniform image segmentation algorithm is used for both investigations [14, 15]. In brief, the Angiovue OCT obtains amplitude decorrelation angiography images using an A-scan rate of 70,000 scans per second, a light source centered on 840 nm and a bandwidth of 50 nm [8]. Each OCT-A volume contains 304 x 304 A-scans with two consecutive B-scans captured at each fixed position before proceeding to the next sampling location. Split-spectrum amplitude-decorrelation angiography is used to extract the OCT angiography information [24, 25]. Motion correction to minimize motion artifacts arising from microsaccades and fixation changes is used. Angiography information is displayed en face as the maximum of the decorrelation values within the corresponding layer. Only images with optimal image quality (signal strength index, SSI>50), no motion artifacts, vitreous floaters, peripapillary atrophy or other artifacts were selected for analysis. The software-provided peripapillary sectors are based on the Garway-Heath map [26]. The RPC layer is defined as the layer between the outer limit of the retinal nerve fiber layer and the internal limiting membrane. The 4.5 mm x 4.5 mm scan size was used. The inner elliptical contour (which defines the optic nerve head) is obtained by automatically fitting an ellipse to the disc margin based on the OCT en face image. The peripapillary area is defined as the area between the inner and outer ellipses. The ring width between the inner and outer elliptical contour lines was 0.75 mm in all cases.

### Optical coherence tomography for retinal nerve fiber layer measurement

Image acquisition was made via undilated pupil with the Angiovue/RTvue-XR Avanti RTVue-XR Fourier-domain OCT instrument (Optovue Inc., Fremont, CA, USA). The working principle and the technical details of image acquisition used by us have been described in detail elsewhere [27]. For RNFLT measurements, the standard glaucoma protocol was used. This includes a 3-dimensional optic disc scan for the definition of the disc margin based on computer-assisted determination of retinal pigment epithelium–choroid end points; an ONH map scan that measures the RNFLT in a zone with a diameter of 4 mm automatically centered on the predefined disc (this process is operator independent). Each ONH scan consists of 12 radial lines and 6 concentric rings that are used to create an RNFLT map. The measuring circle (920 points) is derived from this map after the sample circle is adjusted to be centered on the optic disc. RNFLT is automatically determined for different areas of the 360° measuring circle of 3.45 mm in diameter centered on the disc. This measuring circle is the software-provided standard circle for RNFLT measurement. The software also provides RNFLT values for each of 16 individual sectors of the measuring circle around the optic nerve head. For reference purposes, the sectors, all of equal size (22.5°), are numbered in sequence from the temporal side of the horizontal meridian (clockwise for the right, and anticlockwise for the left eye). Image quality was carefully checked after each image acquisition, and all images of insufficient quality or with any artifact were rejected. Only images with signal strength index (SSI) >50 were used. In the current investigation the RNFLT values provided for the total circle (total peripapillary RNFLT) and 2 of the 16 22.5°-sized sector RNFLT values (the inferotemporal and the superotemporal RNFLT, Fig 1) were used. The RNFLT sectors were determined based on anatomical considerations using the instrument-provided 16 RNFLT sectors [23, 26].

### Visual field testing and determination of the visual field clusters

The same calibrated Octopus 900 perimeter (Haag-Streit AG, Koeniz-Berne, Switzerland) was used for all tests. The G 2 test of the central 30° visual field (phases 1 and 2, which provide doubled threshold determination) was tested with normal (bracketing) strategy within 3 months from the OCT imaging (typically on the same day). Current ametropia was corrected for according to the manufacturer’s recommendation. Artificial tear drops were given before the 5-minute adaptation period. The right eye was tested first in all cases. Only reproducible tests with less than 20% false positive and 20% false negative response rates were used for evaluation. The manufacturer-provided 10 visual field clusters [23] were used (Fig 1). The clusters of the left eyes were mirrored and numbered as those of right eyes. For the cluster structure-function investigation the software-provided uncorrected cluster MD values (in dB) were used. In the normal (bracketing) strategy 4.0 dB luminance steps are used to determine the threshold sensitivity, which is refined using 2.0 and 1.0 dB steps. Of the global indices mean sensitivity (MS) and mean defect (MD) were analyzed. In Octopus perimetry the abnormal MD values are positive numbers.

### Statistics

The raw measurement data are shown in S1 Table. The STATA 6.0 program package was used for statistical analysis. The normality of distribution of the study sample was assessed with the Shapiro–Wilk test. Descriptive statistics are presented as means ± standard deviations for normally distributed variables, and medians and inter-quartile ranges for non-normally distributed variables. Differences between the patient groups were investigated using ANOVA and the Sidak test, and the Kruskall-Wallis test with Bonferroni corrected paired comparison, as appropriate. The relationship between sector PAFD and cluster MD, and sector NFLT and cluster MD was evaluated using quadratic regression. The 95% confidence limits (bias corrected) for the differences between the coefficients of determination (R^2^) were determined with bootstrapping procedures. The relationship between global PAFD and global indices, and total peripapillary RNFLT and global indices, was investigated with Spearman’s correlation. A P-value <0.05 was considered statistically significant.

## Results

Inferotemporal and superotemporal sector RNFLT and PAFD, inferotemporal and superotemporal Octopus perimeter cluster MD, and their comparison between the patient groups are shown in Table 2, respectively. Significant differences were found for all three parameters between the groups (ANOVA P<0.05, Kruskall-Wallis test P<0.05, respectively). The sector RNFLT and sector PAFD values were significantly lower, and the cluster MD values were significantly higher in the glaucoma group than in the healthy and OHT groups, respectively. No difference between the corresponding inferotemporal and superotemporal sector PAFD, sector RNFLT and cluster MD was seen in the total population and the three patient groups, respectively (paired t-test, P>0.05). Table 2 shows the comparison of the corresponding inferotemporal and superotemporal parameters for the glaucoma group.

**Table 2:** Comparison of the sector and cluster values between the groups and the corresponding superotemporal and inferotemporal areas in the glaucoma group, respectively.

	Healthy eyes (1) (n = 13)	OHT eyes (2) (n = 22)	Glaucoma eyes (3) (n = 27)	P-value for comparison between groups	P-value for ST vs. IT comparison, glaucoma group
IT sector PAFD (%; mean ± SD)	61.48 ± 4.48	59.30 ± 3.81	53.0 ± 6.87	1 vs. 2: 0.598* 1 vs.3: < 0.001* 2 vs.3: 0.001*	0.4790^#^
ST sector PAFD (%;mean ± SD)	58.2 ± 4.38	59.2 ± 6.35	51.58 ± 9.60	1 vs.2: 0.977* 1 vs.3: <0.039* 2 vs.3: 0.003*	
IT sector RNFLT (μm; mean ± SD)	127.5 ± 20.7	128.8 ± 16.4	90.0 ± 26.9	1 vs. 2: 0.998* 1 vs.3:<0.001* 2 vs.3: < 0.001*	0.9519^#^
ST sector RNFLT (μm; mean ± SD)	132.6 ± 18.8	133.8 ± 15.6	89.6 ± 23.1	1 vs. 2: 0.998* 1 vs.3:<0.001* 2 vs.3: <0.001*	
IT cluster MD (dB; median, quartiles)	-0.5	-0.3	4.8	1 vs. 2: 0.529‡ 1 vs.3: < 0.001‡ 2 vs. 3: <0.001‡	0.8101^#^
	-1.3 to 0.5	-0.8 to 0.7	1.6 to 18.9		
ST cluster MD (dB; median, quartiles)	-0.4	0.6	5.9	1 vs. 2: 0.999‡ 1 vs.3: <0.001‡ 2 vs.3: <0.001‡	
	-1.0 to 0.8	-0.5 to 1.3	1.8 to 18.4		

OHT, ocular hypertension; IT, inferotemporal; ST, superotemporal; PAFD, peripapillary angioflow-density; RNFLT, retina nerve fiber layer thickness; MD mean defect

*Sidak test

‡ Kruskall-Wallis test with Bonferroni corrected paired comparison

# paired t-test

The relationship between total peripapillary PAFD, total peripapillary RNFLT, and visual field global MS and global MD is shown in Table 3 for the total population, respectively. Total peripapillary PAFD and total peripapillary RNFLT showed similar strong positive correlation with MS; and similar strong negative correlation with global MD.

**Table 3:** Spearman’s correlation coefficients (r), their significance and comparison for global correlations in the total population.

	Global MS (dB)	Global MD (dB)
r-value for total peripapillary PAFD	0.6710	-0.4462
P-value for total peripapillary PAFD	<0.0001	0.0003
r-value for total peripapillary RNFLT	0.6088	-0.4412
P-value for total peripapillary RNFLT	<0.0001	0.0004

MS, mean sensitivity; MD, mean defect; PAFD, peripapillary angioflow-density; RNFLT, retinal nerve fiber layer thickness

**Fig 2:**
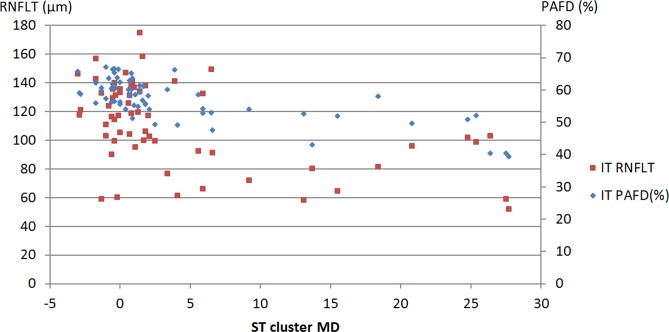
Scatter plot of inferotemporal sector peripapillary angioflow-density (PAFD), inferotemporal retinal nerve fiber layer thickness (RNFLT) and superotemporal cluster mean defect (ST cluster MD) for the total population.

**Fig 3:**
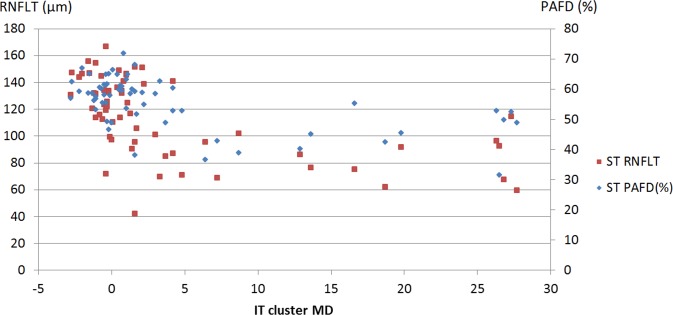
Scatter plot of superotemporal sector peripapillary angioflow-density (PAFD), superotemporal retinal nerve fiber layer thickness (RNFLT) and inferotemporal cluster mean defect (IT cluster MD) for the total population.

The sector—cluster relationships are shown in Table 4 for the total population. The confidence limits of the difference show that the R^2^ value calculated for the relationship between inferotemporal sector PAFD and superotemporal cluster MD (0.5141, P<0.0001) was significantly higher than that calculated for the relationship between inferotemporal sector RNFLT and superotemporal cluster MD (0.2546 P = 0.0001, Fig 2). The R^2^ values for the relationships between superotemporal sector PAFD and inferotemporal cluster MD (0.3747, P<0.0001), and superotemporal sector RNFLT and inferotemporal cluster MD (0.4037, P<0.0001, Fig 3) were similar.

**Table 4:** Differences between the corresponding R^2^ values investigated with bias corrected bootstrapping procedure in the total population.

Sector—cluster pair	R^2^-value	P-value	95% CI
IT PAFD—ST cluster MD	0.5141	<0.0001	0.0153 to 0.4827
IT RNFLT—ST cluster MD	0.2546	0.0001	
ST PAFD—IT cluster MD	0.3747	<0.0001	-0.2394 to 0.1768
ST RNFLT—IT cluster MD	0.4037	<0.0001	

MD, mean defect; PAFD, peripapillary angioflow-density; RNFLT, retinal nerve fiber layer thickness; IT, inferotemporal; ST, superotemporal

## Discussion

In the current investigation we evaluated the relationship between global, inferotemporal and superotemporal peripapillary PAFD, a non-invasive OCT angiography parameter of peripapillary perfusion of the RPC layer, and the spatially corresponding global and cluster Octopus visual field MD values, respectively, in a population comprising healthy, medically controlled OHT and predominantly mild-to-moderate primary open-angle glaucoma eyes. Global MD and the 10 cluster MDs are those Octopus visual field parameters offered by the EyeSuite software for the G test which have been widely used for glaucoma severity classification, glaucoma diagnostics and follow-up in clinical practice [22, 23]. In addition, to establish the relationship between the corresponding sector PAFD and cluster MD values for those sector—cluster pairs which have been established in our earlier study for sector RNFLT and are available with the PAFD analysis software version used by us [14, 15, 23] we also compared the strength of the relationship between the corresponding sector RNFLT and sector PAFD, and the spatially corresponding visual field cluster MD. The background of this comparison was that decreased optic nerve head and peripapillary perfusion has been considered as one potential reason for the development and progression of glaucomatous structural damage, but due to the technical difficulties of reliable, non-invasive and separated measurement of small vessel perfusion in various sectors of the peripapillary retinal nerve fiber layer the potential role of segmental perfusion changes in the pathogenesis of glaucoma could not be definitely established [1, 4, 6, 7, 28]. Both peripapillary PAFD and RNFLT investigated in the current study were measured with the same OCT instrument, in which uniform image segmentation algorithm is used for both investigations, and the definition of the RPC layer (in the angiography mode) and the RNFL layer (in RNFLT measurement mode) is identical [14, 15]. Thus PAFD (the density of the perfused capillaries based on split-spectrum amplitude-decorrelation and expressed in % of the measured RNFL layer in its full thickness) and RNFLT reflect two different aspects of the same tissue layer: the local perfusion and the tissue thickness, respectively. We supposed that if decreased perfusion in a peripapillary sector is a consequence of tissue loss, a similar relationship between sector PAFD and cluster MD, and sector RNFLT and cluster MD will be seen, similarly to the results of the earlier anatomical investigations [20, 21].

The main result of the current investigation is that in addition to the similar strong relationship of global FAFD and global RNFLT with global visual field MS and MD, respectively, for the inferotemporal peripapillary sector the relationship between PAFD and the corresponding superotemporal cluster MD was even stronger than that between RNFLT and the superotemporal cluster MD. Since no eyes with peripapillary atrophy and high myopia, and no images with segmentation error or other artifact were included in the analysis, our results are suggestive for true relationships. It is of interest that no significant difference was seen for the correlations between the superotemporal sector parameters and the spatially corresponding inferotemporal cluster MD. It is also important to note that no significant difference was found between the corresponding inferotemporal and superotemporal PAFD, RNFLT and cluster MD values, respectively; thus the sector-related difference between the correlations cannot be explained with regional differences of disease severity.

Using various methods it has been repeatedly shown for decades that inferior and inferotemporal optic nerve head, lamina cribrosa, neuroretinal rim tissue and RNFLT are particularly susceptible to early glaucomatous damage and glaucomatous progression, and the relationship between inferotemporal RNFLT and the spatially corresponding visual field sensitivity is particularly strong [23, 29–32]. These data suggest that characteristics of the glaucomatous structural damage may be better investigated in the inferotemporal peripapillary sector than in other peripapillary areas. Our results do not provide any explanation for the mechanism of increased susceptibility to glaucomatous damage in the inferotemporal peripapillary sector, and do not prove that impaired peripapillary perfusion has a role in the development of localized glaucomatous structural damage, but they suggest that the inferotemporal sector PAFD may be a useful new indicator of glaucomatous damage associated with localized visual field deterioration.

Our study has limitations. All our patients were white Europeans with low refractive error, and all glaucoma eyes had high pressure primary open-angle glaucoma, thus limited conclusions can be drawn from our results with regard to other ethnic groups, other types of glaucoma and eyes with high myopia or hyperopia. Though the number of eyes was relatively low due to the high image quality criteria we did find strong and significant relationships between the cluster MD values and the corresponding sector PAFD and sector RNFLT values.

Therefore we do not think that the number of eyes represented a limitation in our investigation. Due to the fixed parameters of the Angiovue OCT the size of the corresponding peripapillary RNFLT and PAFD sectors was similar but not exactly the same. Considering the strong relationship between RNFLT sectors and the Octopus clusters [20, 21] this difference worked against finding significant relationships between the sector PAFD values and the Octopus cluster MD values. Still, significant R^2^ values were found for all sector relationships. Due to the technical limitation of the OCT system used by us we could not measure and correct for disc torsion. Since the inferotemporal RNFLT sector is wider to the nasal direction than the inferotemporal PAFD sector a significant inferior disc torsion could considerably influence the result. Our study was a cross-sectional evaluation across a wide range of glaucoma severity, therefore it does not provide information on the relationship between longitudinal change of PAFD and longitudinal change of cluster MD in glaucoma.

In conclusion, in a population comprising healthy, medically controlled OHT and predominantly mild-to-moderate severity primary open-angle glaucoma eyes we found a strong relationship between inferotemporal peripapillary PAFD and superotemporal cluster MD, and a strong relationship between superotemporal peripapillary PAFD and inferotemporal cluster MD. In addition, we found that the R^2^ value calculated for the relationship between inferotemporal sector PAFD and superotemporal cluster MD was even greater than that between inferotemporal peripapillary RNFLT and superotemporal cluster MD. Our results suggest that use of sector PAFD, a new, non-invasive OCT angiography parameter of sector perfusion in the RPC layer, may potentially be used in structure-function investigations and clinical glaucoma research.

## Supporting information

S1 Table Anonymous raw measurement data used for calculation of the relationship between sector peripapillary angioflow density and Octopus visual field cluster mean defect. (XLSX)
